# Supplementary material for: Spatial redundancy transformer for self-supervised fluorescence image denoising
Source: Nat Comput Sci. 2023 Dec 11;3(12):1067–80. doi: 10.1038/s43588-023-00568-2 (PMC10766531; doi:10.1038/s43588-023-00568-2)
Supplement: Supplementary file 2 — Reporting Summary [file 43588_2023_568_MOESM2_ESM.pdf]

nature portfolio

Corresponding author(s): Qinghai Dai; Haoqian Wang; Jiamin Wu

Last updated by author(s): Oct 20, 2023

Reporting Summary

Nature Portfolio wishes to improve the reproducibility of the work that we publish. This form provides structure for consistency and transparency in reporting. For further information on Nature Portfolio policies, see our Editorial Policies and the Editorial Policy Checklist.

Statistics

For all statistical analyses, confirm that the following items are present in the figure legend, table legend, main text, or Methods section.

n/a	Confirmed	
☐	☒	The exact sample size (*n*) for each experimental group/condition, given as a discrete number and unit of measurement
☐	☒	A statement on whether measurements were taken from distinct samples or whether the same sample was measured repeatedly
☒	☐	The statistical test(s) used AND whether they are one- or two-sided<br>*Only common tests should be described solely by name; describe more complex techniques in the Methods section.*
☒	☐	A description of all covariates tested
☒	☐	A description of any assumptions or corrections, such as tests of normality and adjustment for multiple comparisons
☐	☒	A full description of the statistical parameters including central tendency (e.g. means) or other basic estimates (e.g. regression coefficient) AND variation (e.g. standard deviation) or associated estimates of uncertainty (e.g. confidence intervals)
☒	☐	For null hypothesis testing, the test statistic (e.g. *F*, *t*, *r*) with confidence intervals, effect sizes, degrees of freedom and *P* value noted<br>*Give P values as exact values whenever suitable.*
☒	☐	For Bayesian analysis, information on the choice of priors and Markov chain Monte Carlo settings
☒	☐	For hierarchical and complex designs, identification of the appropriate level for tests and full reporting of outcomes
☐	☒	Estimates of effect sizes (e.g. Cohen's *d*, Pearson's *r*), indicating how they were calculated

Our web collection on statistics for biologists contains articles on many of the points above.

Software and code

Policy information about availability of computer code

Data collection	The calcium imaging data were collected by a two-photon microscopes controlled by ScanImage 5.7 (Free release,Vidrio). The single-molecule localization microscopic imaging data were collected by a commercial Nikon N-STORM system equipped with laser sources of 405 nm and 640 nm.
Data analysis	Data simulation and result analysis were performed using custom Matlab (R2019b MathWorks) scripts. All deep learning models reported in this work were implemented with standard libraries of Python 3.6.0) with PyTorch (1.7.0, Facebook). All results of DeepCAD were obtained with the released code (https://github.com/cabooster/DeepCAD-RT). Single-molecule localization was implemented with the open-source ThunderSTORM plugin (v1.3) of Fiji (https://github.com/zitmen/thunderstorm/).The complete code of SRDTrans has been made publicly available at https://github.com/cabooster/SRDTrans.

For manuscripts utilizing custom algorithms or software that are central to the research but not yet described in published literature, software must be made available to editors and reviewers. We strongly encourage code deposition in a community repository (e.g. GitHub). See the Nature Portfolio guidelines for submitting code & software for further information.

Data

Policy information about availability of data

All manuscripts must include a data availability statement. This statement should provide the following information, where applicable:

- Accession codes, unique identifiers, or web links for publicly available datasets
- A description of any restrictions on data availability
- For clinical datasets or third party data, please ensure that the statement adheres to our policy

We have no restrictions on data availability. Both the simulated and experimental data of single-molecule localization microscopy and two-photon calcium imaging in this work are available at https://github.com/cabooster/SRDTrans/tree/main/datasets. The MNIST dataset used for simulation is publicly available at http://yann.lecun.com/exdb/mnist/

Human research participants

Policy information about studies involving human research participants and Sex and Gender in Research.

Reporting on sex and gender	Not relevant.
Population characteristics	Not relevant.
Recruitment	Not relevant.
Ethics oversight	Not relevant.

Note that full information on the approval of the study protocol must also be provided in the manuscript.

Field-specific reporting

Please select the one below that is the best fit for your research. If you are not sure, read the appropriate sections before making your selection.

☒ Life sciences ☐ Behavioural & social sciences ☐ Ecological, evolutionary & environmental sciences

For a reference copy of the document with all sections, see nature.com/documents/nr-reporting-summary-flat.pdf

Life sciences study design

All studies must disclose on these points even when the disclosure is negative.

Sample size	The sample size(n) of each experiment is provided in the figure/table legends in the main manuscript and supplementary information files.
Data exclusions	No data was excluded from the analysis.
Replication	The number of repetitions for each experiment is provided in the figure/table legends in the main manuscript and supplementary information files.
Randomization	Not relevant, as there were no such experimental groups in this study.
Blinding	Not relevant, as there were no such experimental groups in this study.

Reporting for specific materials, systems and methods

We require information from authors about some types of materials, experimental systems and methods used in many studies. Here, indicate whether each material, system or method listed is relevant to your study. If you are not sure if a list item applies to your research, read the appropriate section before selecting a response.

Materials & experimental systems

n/a	Involved in the study
☐	☒ Antibodies
☐	☒ Eukaryotic cell lines
☒	☐ Palaeontology and archaeology
☐	☒ Animals and other organisms
☒	☐ Clinical data
☒	☐ Dual use research of concern

Methods

n/a	Involved in the study
☒	☐ ChIP-seq
☒	☐ Flow cytometry
☒	☐ MRI-based neuroimaging

Antibodies

Antibodies used	Primary antibody: mouse anti-Beta-tubulin E7 (AB_2315513, E7, DSHB).<br>Secondary antibody: AffiniPure Donkey Anti-Mouse IgG (AB_2347508, 715-005-150, Jackson Immuno Research) conjugated with Cy5 (PA25001, GE Healthcare).
Validation	The antibodies have been extensively used in previous studies and the the statements related to antibody validation can be found at manufacturer' website(mouse anti-Beta-tubulin E7: https://dshb.biology.uiowa.edu/E7_2; AffiniPure Donkey Anti-Mouse IgG: https://www.jacksonimmuno.com/catalog/products/715-005-150). Labeled samples were purchased from Standard Imaging Company (https://www.standardimaging.cn/standardsample?lang=en).

Eukaryotic cell lines

Policy information about cell lines and Sex and Gender in Research

Cell line source(s)	The Biologics Standards-Cercopithecus-1 (BSC-1) cell line purchased from Pricella Life Technology Co. LTD was used for STORM imaging.
Authentication	None of the cell lines were authenticated.
Mycoplasma contamination	Cell lines have been tested and were negative for mycoplasma contamination.
Commonly misidentified lines<br>(See ICLAC register)	No commonly misidentified lines were used.

Animals and other research organisms

Policy information about studies involving animals; ARRIVE guidelines recommended for reporting animal research, and Sex and Gender in Research

Laboratory animals	For calcium imaging of neural circuits in the mouse brain, we used transgenic mice (male or female) hybridized between Rasgrf2-2A-dCre mice and Ai148 (TIT2L-GC6f-ICL-tTA2)-D mice expressing Cre-dependent GCaMP6f genetically encoded calcium indicator (GECI). All mice were aged 8-12 weeks and were housed in cages (24°C, 50% humidity) in groups of 1-5 under a reverse light cycle.
Wild animals	None
Reporting on sex	None
Field-collected samples	None
Ethics oversight	All experiments involving animals were performedin accordance with the institutional guidelines for animal welfare and have been approved by the Animal Care and Use Committee of Tsinghua University.

Note that full information on the approval of the study protocol must also be provided in the manuscript.
